# Doing landscape: sensorial and artistic approaches to Donkalnis and Spiginas Mesolithic–Neolithic ritual sites in western Lithuania

**DOI:** 10.1080/1751696X.2024.2338055

**Published:** 2024-04-24

**Authors:** Marja Ahola, Katri Lassila, Kristiina Mannermaa

**Affiliations:** a Department of History Culture, and Communication, University of Oulu, Oulu, Finland; b Department of Cultures, Archaeology, University of Helsinki, Helsinki, Finland; c School of Arts, Design and Architecture, Department of Film, Television and Scenography, Aalto University, Espoo, Finland

**Keywords:** Mesolithic, Neolithic, hunter-gatherers, sensory landscape, ritual deposits, non-human agents

## Abstract

During the Mesolithic and Neolithic, foragers dwelling in the Eastern Baltic, Scandinavia and Fennoscandia regions buried some of their dead on lake islands or other coastal sites. Based on ethnographic accounts, these sites are often understood as liminal places where water separates the lands of the dead and the living. In this paper, we take a more relational view of place and suggest that a particular combination of spatial perception of landscape and the dynamic nature of coastal sites might have contributed to the social agency of these places, resulting in their use as places for ritual activity. By exploring two Mesolithic–Neolithic burial places, Donkalnis and Spiginas (western Lithuania), with sensory archaeological and artistic approaches, we suggest that the ancient foragers of this region buried human bodies in these locations to be part of the place itself. Similar to other depositional acts, this could have been done to mark the location or communicate with the surrounding world.

## 1. Introduction

More than 8000 years ago, forager peoples living on the shores of ancient Lake Biržulis (western Lithuania) visited two small, peculiar-looking islands, Spiginas and Donkalnis, and buried altogether approximately 20 people on the highest points of these sites (Butrimas 2012, 2019). The buried were women, men and children who were mostly interred individually. As in other forager burial sites in nearby regions (Ahola 2019; Gurina 1956; Larsson 1989; Tõrv 2016; Zagorskis 2004), the buried bodies were adorned with personal ornamentation and in some cases with red ochre. The initial burials were followed by others, but at intervals of several hundred or even several thousand years (Table 1), suggesting that not all members of the community were buried here. On the contrary, it seems likely that a multiplicity of mortuary places and practices coexisted (Nilsson Stutz 2014). However, as newer burials at Spiginas and Donkalnis did mainly not interfere with the older ones (Butrimas 2012), it seems that the islands, along with the ritual deposits of human bodies, were remembered over a long period.

**Table 1.** Summary of Donkalnis and Spiginas burials and their datings.

Grave no.	Radiocarbon dating (BP) and dated material	Calibrated date, 95% probability	Period	Description	References	Notes
**Donkalnis**						
1	4610 ± 35; 4399 ± 42 (human remains)	3516–3191; 3323–2907	Subneolithic	A burial of a woman ∼20 years old. No artefacts or other finds. The grave pit was marked by small reddish ochre stains	Butrimas (2012, 2019), Simčenka et al. (2022)	
2	7405 ± 45 (human remains)	6400–6110	Mesolithic	A burial of a man ∼25–30 years old covered with intensive ochre. 57 animal tooth pendants were discovered from the head region of the deceased, and a small flint blade on top of the skull	Antanaitis-Jacobs et al. (2009), Butrimas (2019, 2012)	A hearth with knapped stones and ochre next to the burial
3	5785 ± 40 (human remains)	4730–4530	Subneolithic	A burial of a woman ∼25–30 years old with small amounts of ochre. No grave goods or other finds	Antanaitis-Jacobs et al. (2009), Butrimas (2019, 2012)	Despite a hiatus of several thousand years, the burial was located next to burial 2
4	6995 ± 65 (human remains); 5970–5740 (elk incisor)	6000–5740;5970–5740	Mesolithic	A partly destroyed burial of a man aged 50–60 years accompanied with more than 80 tooth pendants and ochre	Antanaitis-Jacobs et al. (2009), Butrimas (2012, 2019), Piličiauskas and Heron (2015)	
5	7140 ± 40 (human remains)	6075–5920	Mesolithic	A burial of a child ∼7 years old associated with several animal tooth pendants and a bone arrowhead	Butrimas (2012, 2019), Piličiauskas et al. (2017)	
5	7110 ± 40 (human remains)	6060–5900	Mesolithic	A burial of an infant positioned next to the above described burial	Butrimas (2012, 2019), Piličiauskas et al. (2017)	
6	5770 ± 40 (human remains)	4720–4530	Subneolithic	A partly destroyed burial of a woman aged 35–40 years. No ochre, artefacts or other finds	Butrimas (2012), Piličiauskas et al. (2017)	
7	4674 ± 29 (human remains)	3521–3371	Subneolithic	A poorly preserved burial of a man aged over 50 years. A triangular flint arrowhead, a bear mandible, and a bear tooth were discovered next to the feet of the deceased	Butrimas (2012), Simčenka et al. (2022)	
I	5760 ± 40 (human remains)	4712–4502	Subneolithic	Loose human bones from a destroyed grave of an adult man	Simčenka et al. (2022)	
II	5977 ± 40 (human remains)	4988–4730	Subneolithic	Loose human bones from a destroyed grave of an adult woman	Simčenka et al. (2022)	
III	5679 ± 39 (human remains)	4654–4371	Subneolithic	Loose human bones from a destroyed grave of an adult man	Simčenka et al. (2022)	
IV	5822 ± 40 (human remains)	4787–4551	Subneolithic	Loose human bones from a destroyed grave of an subadult with an unknown sex	Simčenka et al. (2022)	
V	6244 ± 43 (human remains)	5312–5059	Late Mesolithic	Loose human bones from a destroyed grave of a man with an unknown age	Simčenka et al. (2022)	
VI	5515 ± 39 (human remains)	4448–4266	Subneolithic	Loose human bones from a destroyed grave of a child with an unknown sex	Simčenka et al. (2022)	
**Spiginas**						
1	5470 ± 40 (human remains); 5370 ± 40 (ungulate long bone)	4440–4240; 4330–4060	Subneolithic	A partly destroyed burial of an adult man aged between 35 and 45 years buried in a pit marked by botches of ochre. Two transverse arrowheads were discovered from the pit along with a small, ochre-stained natural stone	Butrimas (2012), Piličiauskas and Heron (2015)	
2	3580 ± 60 (human remains)	2130–1750	Neolithic; EBA	A burial of a man ∼50–55 years old in a crouched position. No ochre, artefacts or other finds	Butrimas (2012), Piličiauskas et al. (2017)	
3	7780 ± 65 (human remains)	6800–6460	Mesolithic	A poorly preserved burial of a woman with an unknown age. No artefacts or other finds	Butrimas (2012, 2019), Antanaitis-Jacobs et al. (2009)	
4	7470 ± 60 (human remains)	6440–6230	Mesolithic	A well-preserved burial of a woman aged 30–35 years marked by heavy ochre (especially in the head and pelvis regions). The burial was associated with two flint arrowheads and several animal tooth pendants	Butrimas et al. (1985), Butrimas (2012, 2019)	Date obtained with beta decay radiocarbon method

Where possible, radiocarbon determinations represent dates obtained using the conventional accelerator mass spectrometry method.

Considering that the communities dwelling in the ancient Lake Biržulis region returned to Spiginas and Donkalnis repeatedly, there must have been something about these places and their surroundings that made them meaningful to the ancient foragers. Although the inhumations were not as visible as, for example, cairns or burial mounds, the continuous use of the sites suggests that the small islands were nonetheless recognized as ‘special’ or ‘sacred’ – a phenomenon that might be rooted in the perception of landscape. Indeed, several scholars (e.g. Anttonen 2003; Äikäs 2015; Bradley 2000; Jordan 2008; Rainio et al. 2017) have argued that prehistoric and historical communities of the Northern Hemisphere have commonly set apart topographically and sensorially anomalous sites and marked these places with distinctive deposits, caching or creation of rock art to express and reproduce the relationships that link the human collective with the surrounding ecology. For example, the sacred sites of the Sámi people, the *sieidi*, are often located in places with unusual topographic features and accompanied, for example, by echoes or the silence of still water (e.g. Äikäs 2015; Bradley 2000). These sites were visited with offerings of, for example, fish, game, alcohol and small personal objects that were placed in certain locations selected based on the direction from which the *sieidi* was approached (Äikäs and Tolonen 2016). Although no temples or shrines were built, the sensorial perception of the landscape, along with memories of the conducted ritual practices, gave the sites a sacred character in the minds of the people.

Although we cannot assume that the Mesolithic peoples shared a cosmology with the historical forager and pastoralist communities of the Northern Hemisphere and, accordingly, that all hunter-gatherer communities are the same (see Elliott and Warren 2023), striking similarities between the Sámi *sieidi* sites and Mesolithic–Neolithic rock art sites of northern Europe have nevertheless been noted. Indeed, in many cases, the rock art sites are also located on or near special topographic features – on impressive cliffs that might take an anthropomorphic form, by rapids or in sites with adjoining echoes (Anttonen 2003; Damm 2021; Helskog 1999; Lahelma 2008; Rainio et al. 2017). In some cases, the same cliffs have been visited and marked for millennia if not even longer (Seitsonen 2015), suggesting shared ground with the ritual practices conducted at burial sites. In this sense, it is interesting that some scholars (Borić 1999; Conneller 2013; Grünberg 2000) have also noted that prehistoric hunter-gatherer burial sites are occasionally located in somehow anomalous landscapes – in caves, by lagoons or next to large boulders or smooth bedrock. In a previous paper (Ahola 2017), one of the authors suggested that, similar to *sieidi* and rock art sites, these topographic features stand out from the surrounding landscape and could thus act as focal points or liminal boundaries between the world of the living and that of the dead.

Here, it must be noted that many Mesolithic burial sites in northern Europe are located on lake islands (Butrimas 2012; Gurina 1956; Zagorskis 2004). Drawing from ethnographic materials, Adomas Butrimas (2012, 143–146) proposes that the tradition of burying the dead on an island could relate to a worldview in which the world of the dead is separated from the world of the living by a water body. Although this is a plausible explanation especially with regards to historical traditions in which the dead were buried on islands to keep them from returning to the world of the living (Herva and Ylimaunu 2014; Núñez 2015), the Stone Age forager communities likely perceived the world differently, seeing the world as something that was constantly moving, changing and coming into being (Herva and Lahelma 2020, 6–11). In such a relational worldview, the relationship between humans, animals and natural elements was more interactive, and other-than-human agents likely had an agency of their own (Harrison-Buck and Hendon 2018; Herva and Lahelma 2020). Consequently, aside from considerations of the cosmos, the agency of a place might also have played a key role when different locations were chosen for ritual activities. Indeed, as Melissa Baltus (2018, 86) explains: ‘Places are agential and able to gather human and other-than-human persons, forces, elements, deities, and other social agents that were entangled within social relationships, past or present. These are places at which diverging histories and identities may converge for particular moments. Additionally, these are places where worlds may converge, bringing humans into contact with other-than-human agents. These places themselves can be considered to be social entities, having personalities of their own through particular combinations of sounds, smells, viewsheds, and activities’.

Considering that the creation of rock art, caching and depositional acts are seen as ways of communicating with other-than-human agents, it is plausible that human burials were performed for the same purpose. In other words, human burials would not be ritual responses to death, but rather communication with places that were considered social entities. If so, could it still be possible to trace the multisensory attributes that gave these places their own agency? The idea is intriguing as it suggests that we should change the perspective from seeing the landscape of the ancient burial sites solely as ‘deathscapes', that is, landscapes with death connotations (Dimakis 2015; Maddrel and Sidaway 2016), to the agency of natural elements, that is, the multisensorial experience of a landscape. In other words, we could further investigate ancient burial sites by focusing on landscape attributes that might set them apart from the surrounding landscape and, accordingly, give them the agency that demanded action.

**Figure 1.**
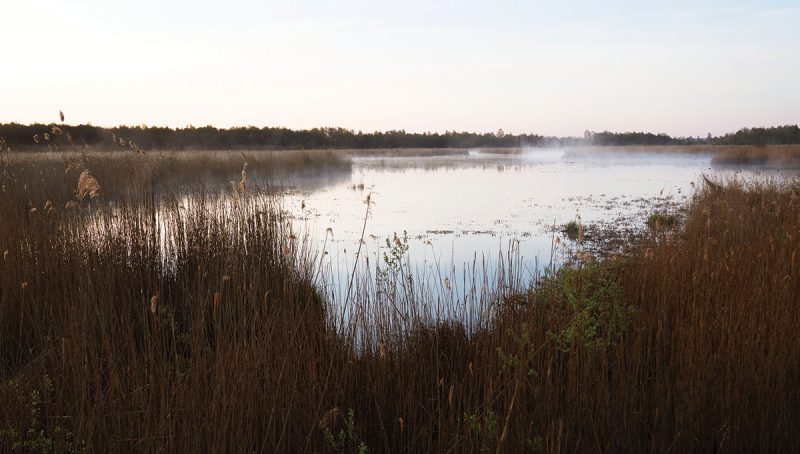
The remnants of ancient Lake Biržulis photographed early in the morning. Photograph: K. Lassila.

However, even though it is likely that Donkalnis and Spiginas were experienced and given meanings via multiple senses, an analysis including sounds and smells is impossible due to the changes that the landscape and vegetation have undergone during the past 8000 years. In addition to changes in climate and water levels, the most prominent change is that Lake Biržulis dried up in the mid-twentieth century (Butrimas 2012, 29–36). The absence of water changes not only the landscape but also the sound and smellscapes of the ritual sites. Both sites can now be approached by walking instead of by boat, making the kinetic experience different. Nonetheless, as the ancient lake is visible, for example, in the early-morning mist that rises from the ancient lake bottom and the scarce remains of the lake itself (Figure 1), it is possible to obtain a glimpse of the ancient landscape. Moreover, as Christopher Tilley (1997) notes, the bones of the land, such as hills, rocks and valleys, remain substantially the same and thus enable the use of personal spatial experiences when observing the patterned relationships between the site and its setting. Although Tilley’s approach has been criticized as a Western, subjective and generalist approach that does not take into account human variability, for example, how different genders and age groups experience the landscape aside from visual sense (e.g. Brück 2005; Fleming 1999; Nyland 2020b), it is important to note that perception and cognition are grounded in the body and its movement (Ingold 2004). Indeed, by exploring movement and perception from the perspective of Norwegian Stone Age forager rock art, Nyland and Stebergløkken (2020) suggest the following: It seems apparent that variations evoked by viewing the figures at different times, or from different vantage points, may have mattered more than seeing everything at once. Adding varying social context, sites are never static galleries, but the motifs are active players together with humans, movement, time and light.

In other words, walking to, from and around a particular location at different times of the day is important to the way the site is perceived and sensed.

The vision and visibility of the landscape are pivotal for a sense of place in many communities around the world (Damm 2021; Hoaen 2020; Schülke 2020; Van Dyke et al. 2016). For example, Stone Age rock art in northern Fennoscandia is nearly always located near water, at places one would naturally pass or, as mentioned earlier, at locations that draw people to them (Damm 2021). At the same time, the presence of particular rocks, shifting light or the pareidolia effect might have caught the eyes of ancient foragers (Ahola and Lassila 2022; Lahelma 2008; Nyland and Stebergløkken 2020). As many of these sites are also located at transitions in landscapes, often close to river mouths, the sites might have acted, on the one hand, as spiritual places and, on the other, as seasonal and geographical meeting places. Accordingly, even though visualism is often seen as central to modern Western culture (Skeates and Day 2020, 2–3), the way the landscape was visually perceived also seems to have been important for the sense of place and conceptions of the cosmos in Mesolithic–Neolithic Northern Europe.

To explore ‘the bones’ of the Spiginas and Donkalnis landscape, in this paper we combine a sensory archaeological approach to the landscape with artistic methods based on movement and visual perception. As our team consists of archaeologists specializing in the Stone Age (M.A. and K.M.) and a professional photography and video artist specializing, on the one hand, in non-Western landscapes and, on the other, in the way water, sky and land connect within these landscapes (K.L.), we are interested in exploring whether the artist, coming from outside the archaeological tradition, would pay attention to the same landscape features as the archaeologists and whether these attributes could be the key to understanding the significance of the place. From the perspective of artistic tradition, the approach is grounded in a method called artistic research, in which the artist’s subjective perception and practice play a vital role in the formation of results that are made visible in words (Borgdorf 2012). As Borgdorf (2012, 37–38) explains, ‘Artistic practice itself is both an essential part of the research process and the research outcomes’.

Following the core idea of artistic research, we hypothesized that engaging with the landscape via artistic practices would allow us to slowly observe and perceive the landscape from several angles, and accordingly bring forth key aspects of the ancient landscape. At the same time, the artistic practices would create yet another multisensorial layer of ‘being in the place’ consisting of the experience of drawing, photographing and videoing that we could also reflect upon. In other words, we would not only perceive and experience the landscape, but do the landscape. Indeed, even though we cannot escape our own embodied experience of the world, we can nonetheless broaden our understanding of our experiences by reflecting on them with others (Tilley 1997, 2020). Accordingly, the images we created act, on the one hand, as the documentation of the site and its surroundings and, on the other, as the materialization of ‘sensuous scholarship’ (see Skeates and Day 2020, 13–14), which we could compare and discuss after the fieldwork. Here, we also loosely follow the idea of Hamilakis et al. (2009), who suggest that photography production can be understood as something that ‘occupies the space between artwork and ethnographic commentary or intervention’. With this idea, Hamilakis et al. (2009, 289–290) mean that photographs are not mere visual documentation but can also lead to unexpected associations along with the remnants of the continuous biography of a site. However, as we were not working with modernist archaeological ethnography but, rather, with landscape archaeology of the deep past, we did not consider the photography production ethnography but, rather, another method of capturing our sensory experiences of the ancient landscape.

In this paper, we adopt a relational view of place and landscape and hypothesize that ritual deposits in the form of human burials were made at the Spiginas and Donkalnis sites because these locations were considered social agents. Our point of departure is, on the one hand, in the way forager peoples of the Northern Hemisphere understand and perceive landscapes and, on the other, in the way the archaeological evidence of ritual depositions is located within the ancient Lake Biržulis region. Accordingly, through the sensory perception of the landscape at these sites, accompanied by artistic research performed via photography and video art, we investigated whether there was something about these places that could have set them apart from their surroundings as distinctive locations during their period of use, and furthermore whether any remnants of that distinctiveness would still be perceptible after thousands of years.

## 2. The Spiginas and Donkalnis sites

**Figure 2.**
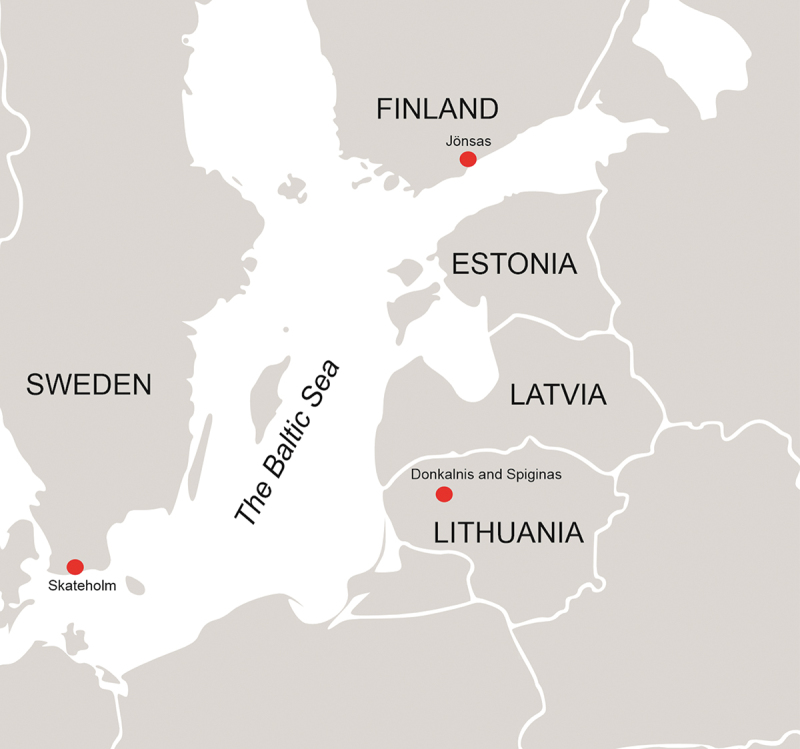
Sites mentioned in this study. Map by M. Ahola. Background map: Wikimedia Commons.

Before moving on, a few words about the sites themselves are appropriate. The Donkalnis and Spiginas Mesolithic–Neolithic burial sites are located in western Lithuania (Figure 2). Both are forager burial sites with the earliest burials dating to the seventh millennium BCE (Table 1). During this time, both sites were small islands located in ancient Lake Biržulis, marked by high water levels and large amounts of fish (Butrimas 2012, 16–19). Although both sites are located in the close vicinity of settlements or campsites with a Stone Age date (Butrimas 2012, Fig. 17), it is unclear whether the settlement sites were in use at the same time as the burial sites. Since remains of other ritual activity (e.g. sacrificial hearths and ochre) have been discovered from a Neolithic settlement site close to Donkalnis, these sites could nonetheless be connected (Butrimas 2019, 204).

**Figure 3.**
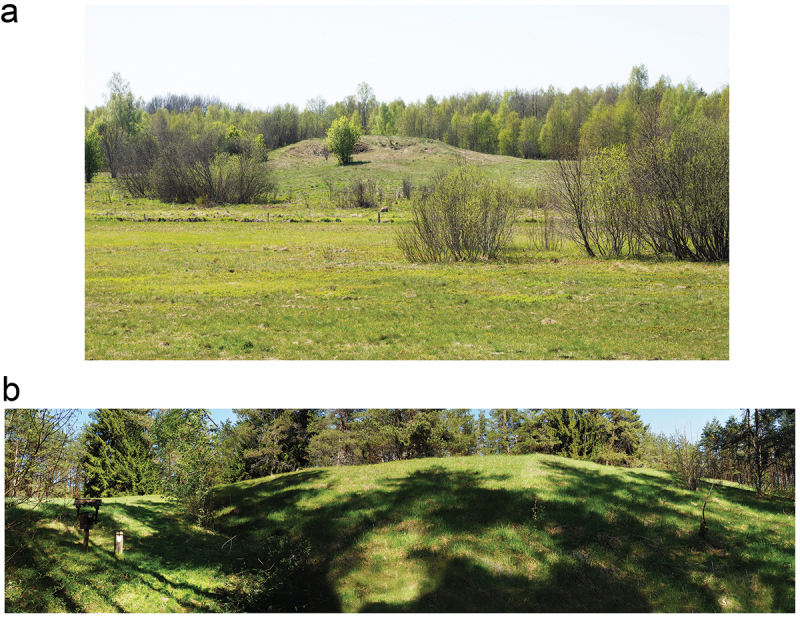
A. The former Donkalnis Island photographed from an ancient shoreline located north of the site. B. An overview of the Spiginas hill compiled from five different photographs. Photographs: K. Lassila.

Geography wise, Donkalnis and Spiginas are former kames, glacial landforms shaped by meltwater, and have commonly been described as ‘loaf-shaped’ (Butrimas 2012, 23–28; 2009, 194–195). Despite having the same basic shape, Donkalnis is clearly flatter, with a height of approximately five to six metres (Figure 3a), while Spiginas is marked by an impressive hill that is situated roughly in the centre of the ancient island (Butrimas 2012, 29–32) (Figure 3b). The human burials at both places were made mainly at the highest points of the islands (Butrimas 2012, Figs. 14 and 47): at Spiginas, all four burials known from the site were found on top of an impressive hill; while at Donkalnis, one burial with a total of 13 inhumations was made on a lower terrace, while the rest were located at the highest point of the ancient island.

The Donkalnis burial site was discovered in the early 1980s by Butrimas (Butrimas 2012, 2019), who excavated seven inhumations from the site. Further remains of six individuals were discovered in the walls of a gravel pit that had destroyed part of the burial site. Most of the inhumations date to the early fifth millennium BCE (Table 1). In addition to these burials, sporadic graves dating to the late seventh millennium BCE, early sixth millennium BCE and mid/late fourth millennium BCE are known from the site. Accordingly, it seems that inhumations were made at the sites only rarely and with a hiatus of several hundred years between burials. As noted previously (Piličiauskas et al. 2017, 534; see also Zagorska 2006; Ahola 2017), this phenomenon is in line with other fisher-hunter-gatherer burial sites in northeastern Europe and the Eastern Baltic. However, in the case of Donkalnis, it is interesting to note that the fourth millennium BCE burial activity coincided with transgression of water levels (Butrimas 2019, 195).

Similar to the Donkalnis site, the Spiginas site was also discovered in the early 1980s and excavated by Butrimas (2012, 2019). In terms of burial activity, the Spiginas site is clearly smaller than the Donkalnis site, and to date only four inhumations have been found at the site. Two of these burials date to the seventh millennium BCE, one to the later part of the fifth millennium BCE and one to the late third millennium BCE (Table 1). Accordingly, it seems that the Spiginas burial ground was used even more rarely than the Donkalnis site. Nevertheless, despite the hiatuses in the burial activity, the site seems to have been remembered or rediscovered repeatedly. In this light, it is interesting that traces of Neolithic and Bronze Age settlements or other activities are known from the slopes of the kame (Butrimas 2012, 83). In this sense, the site is reminiscent of the Jönsas burial site in southern Finland, where the Bronze and Early Iron Age communities of the region reused the ancient cemetery for feasting (Äikäs and Ahola 2020; Purhonen and Ruonavaara 1994). In other words, the place was likely considered somehow special even after the tradition of making a burial was seized. This could also have been the case with Spiginas.

## 3. Materials and methods

The research material for this study was collected mainly during a field trip to western Lithuania in early May 2023, when we visited and documented both sites with photographs, videos, field notes and drawings. The documentation was complemented by written (Butrimas 2012, 2019) and oral descriptions of the sites, along with maps, aerial photographs and geological information published in Butrimas (2012).

### 3.1. Sensory approach

**Figure 4.**
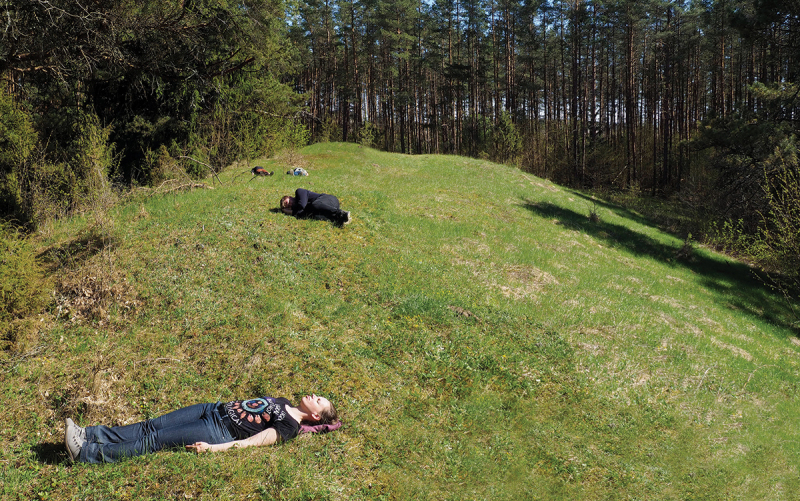
Participants of the field excursion marking the places of Spiginas inhumations. Photograph: K. Lassila.

The first part of our analysis consisted of site visits with the lead excavator, Professor Adomas Butrimas (Vilnius Academy of Sciences). Although we had already carefully studied publications concerning the sites and the human burials (Antanaitis-Jacobs et al. 2009; Butrimas et al. 1985; Butrimas 2012, 2019; Piličiauskas and Heron 2015; Simčenka et al. 2022), being in place with the person who had excavated the sites brought important new information to light. For example, Professor Butrimas was able to point out the places of the burials at the sites. Although these features had been documented and published in site drawings (Butrimas 2012, Figs. 4, 46, 47 and 49), positioning the burials at the sites improved our spatial perception of the ancient island and its ritual deposits (Figure 4). As Tilley (1997, 75) rightly points out: ‘Looking at the two-dimensional plane of the modern topographic map with sites and monuments plotted on it, it is quite impossible to envisage the landscape in which these places are embedded. The representation fails, and cannot substitute for being there, being in place’.

Another key factor in site visits with the lead excavator was the tacit knowledge of the sites and conducted excavations. For example, when recalling the excavations at the Kalniškiai peninsula during the early 1980s, Professor Butrimas explained: ‘When I was excavating this settlement, I often wondered what was on that loaf-shaped hill that was located opposite to the site. I was intrigued by the hill and decided to do some test pitting’ (Butrimas, pers. comm.). The ‘loaf-shaped’ hill was the Donkalnis site, and the test pits brought to light the first inhumations. Accordingly, it was the perception of a topographic anomaly and its spatial relationship to a known Stone Age site that resulted in the discovery of the ritual site. Interestingly, soon after the discovery of the Donkalnis site, a local farmer approached Professor Butrimas and explained that he knew of another site with a very similar hill (Butrimas, pers. comm.). As initial excavations at this hill resulted in the discovery of the Spiginas site, the personal spatial experiences of landscape by different people from different backgrounds have proven to be a fruitful point of departure in locating Mesolithic–Neolithic burial sites. However, Professor Butrimas noted that further excavations at other kames located in the region did not bring forth further burial sites (Butrimas, pers. comm). Accordingly, not all ‘loaf-shaped’ hills were chosen for ritual deposits.

After receiving the crucial background information, we moved to the second part of our sensory approach, in which we aimed to combine Tilley’s (1997, 74) phenomenological method, in which a site is approached slowly from different directions, with artist-researcher Arlander’s (2012) approach, in which repeated encounters with the living environment are transformed into a kind of mediation practice in which the environment and the observer are in close interaction. As Arlander (2012, 16) explains: By returning to the same place once a week for one year and performing the same action in front of a camera that is placed in the same position, and then editing the takes, the slow processes in the environment can be condensed and sped up in the video work. For the performer the act of returning to a site repeatedly produces almost the opposite effect, an extended encounter with the living environment, a kind of meditative practice. Inevitably this way of creating ‘mementos of moments’ in a landscape also means producing more inanimate objects, turning experiences of the living environment into commodities, into video works.

Interestingly, exploring the method from the perspective of drawing, Ville Lukkarinen (2015, 17–19) noted that drawing places creates a sensory relationship with the environment. At the same time, observation through drawing slows the pace and immerses one in the place multisensorially, while the result – the drawing itself – is the materialization of one’s sensorial encounter with the landscape. As Tilley (1997, 75) also proposes that a landscape requires time and repeated visits before the experience is heightened and the locale becomes truly visible, the artistic and archaeological ways of perceiving and engaging with landscape have common ground that relates to time, immersion and revisits to the same locations. However, it must be noted that our short field trip to Lithuania did not allow us to visit the sites on a weekly basis or during different seasons. Instead, we spent approximately four hours at Donkalnis and visited Spiginas on two different days and at different times of day: first in the afternoon and second in the early morning, spending several hours at the site on both occasions. In this sense, our analyses from these sites should not be understood as comparative, but rather as case studies that test the artistic phenomenological approach to an archaeological site.

**Figure 5.**
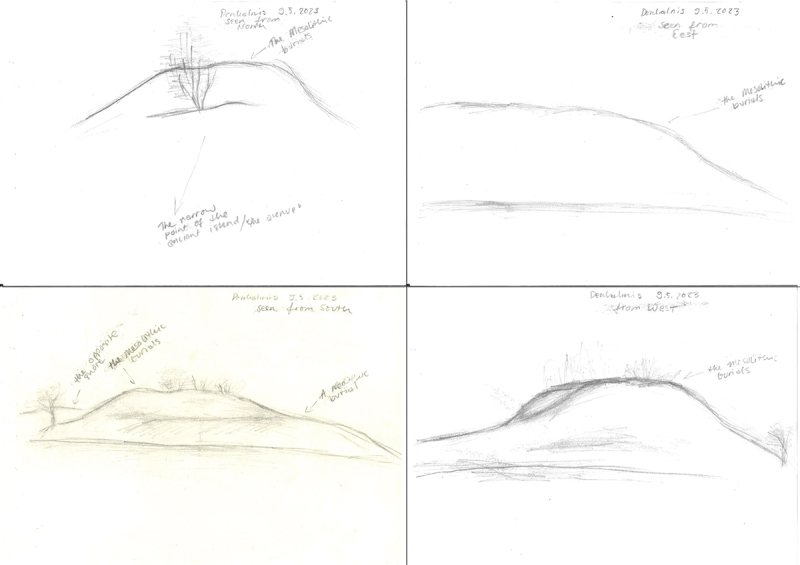
Drawings representing ancient Donkalnis Island from different directions. Illustration: M. Ahola.

In practice, we (M.A. and K.M.) conducted our analysis by approaching the sites from several different directions, perceiving the surroundings of the site from the site and slowing down by drawing the site and writing notes (Figure 5). In other words, our methods consisted of both the perception and experience of the place grounded in movement (Ingold 2004; see also Nyland and Stebergløkken 2020) and in slowing pace and immersion in the landscape. As non-professionals, our drawings were meant to be not art but, rather, a way to ‘do the landscape’. As explained earlier, we also used these ‘mementos of moments’ (Arlander 2012, 16) to recall and reflect upon our spatial experiences of the landscape. Although the approach worked well in the case of Donkalnis (Figure 5), the Spiginas site was located in a dense forest that made visual perception and documentation by drawing difficult. Accordingly, in the case of the Spiginas site, we relied more on written documentation of our phenomenological walks, combined with photography and videography (Supplementary material 1–3^1^).

### 3.2. Artistic approach

Photography and videography played a significant role in the artistic process. As Hamilakis et al. (2009, 285) suggest, there is much to be gained in exploring the connections between photography and archaeology, as they share several ontological and epistemological features. Indeed, much like photography, which offers an interpretation removed from the world it represents (see e.g. Sontag 1979, 28), archaeology objectifies its subject of research (Hamilakis et al. 2009). However, the investigation of the intersections between archaeology and photography can be taken even further, especially in the context of burial sites. For example, André Bazin (2008) refers to photography as a form of mummification, wherein the captured subject matter remains unchanged over time, much like an insect preserved in amber. According to Bazin’s interpretation (Bazin 2008, 14), photography and death are closely intertwined, as photography halts life and change. Numerous others have similarly juxtaposed photography and death (e.g. Barthes 1986, 83; Bellour 2007, 119; Mulvey 2006). The notion of stillness and detachment from time is also inherent in the context of photography (see e.g. Lassila 2023, 92–94). Even though the photographic image might be considered an objectified and detached representation of the subject, the artist’s experience during the act of capturing the image and, thereby, the process of image creation can be powerful and, at its best, immersive, conscious and highly sensory.

**Figure 6.**
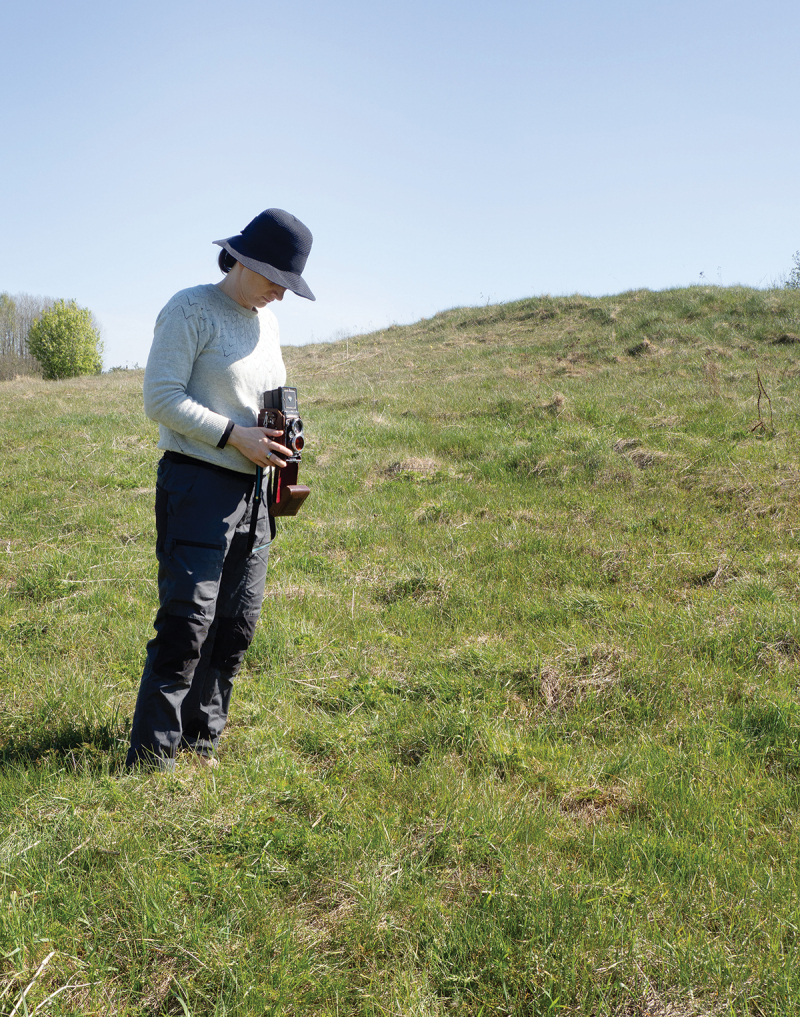
Katri Lassila photographing with the Rolleiflex in Donkalnis. Photograph: K. Lassila.

In both areas of study, we delved into landscapes using an immersive approach within artistic research. K.L. adopted a method inspired by Arlander’s approach outlined in the article ‘Performing and Thinking with Trees’ (Arlander 2022, 21), which advocates for a posthumanist perspective that urges a re-evaluation of our understanding of landscapes. This perspective emphasizes that the environment comprises entities and life forms with varying degrees of agency. Arlander’s starting point in the project was the agency of trees as components of the landscape, after which K.L. tailored her own method for the purposes of this research. The approach involved a sensory and embodied engagement with the landscape and its elements and employed various photographic techniques during artistic practice at these sites. This encompassed alternating between capturing moving digital images and digital still images and using two distinct analogue cameras: the square-format Rolleiflex and the panoramic Horizon camera (Figure 6).

The photography sessions were conducted over periods of a few hours each, during which the photographic approach was varied (Supplementary material 1). The sites evoked two markedly distinct sensory experiences. At Donkalnis, the scorching midday sun had turned the coarse grass a sharp yellowed hue. Nettles grew on the ground. The landscape appeared tranquil and drowsy, swiftly inducing a sense of languor. The earth emitted smells of heat and aridity. The prevailing ambiance was serene and motionless. In the distance, the calls of cranes could be heard. The site’s circular and island-like form, distinct from its surroundings, was easily discernible at ground level (Supplementary material 1–2).

At Spiginas, the majority of the photography took place just after sunrise (Supplementary material 1). Encircled by the forest, the hillock was still moist from the night’s dew, and the sun had yet to rise above the treetops, leaving the ground in shadow. The air was cool and damp from the night and full of the rich scents of earth and grass. Small insects moved across the ground. From the nearby lake and reeds, the repeated calls of waterfowl echoed. The cool ground quickly started to feel cold. From ground level, the oval shape of the place rose nearly vertically towards the sky, resembling the back of a large creature. Additionally, the reed-covered edge, situated close to the burial site and marking the former and partially drained shoreline of the ancient lake, held visual fascination. The sunrise from the lake produced striking patterns of light and shadow on the grassy shore as the sun’s rays filtered through the mist and the tree trunks growing along the shore. The visual impact of the shoreline, coupled with the rising, steep terrain in the background, undoubtedly constituted a significant landscape view in the area prior to its reforestation (Supplementary material 1 and 3).

## 4. Results and discussion

Following the encounters with these two disparate landscapes and locations, we analysed the impressions they evoked. Although we had decided to focus solely on visual and spatial perceptions, the landscape was nonetheless rendered perceptible through multiple senses – sight, hearing, smell and touch. Here, it is noteworthy that many of these aspects relate to our sense of place in the modern world where the Spiginas and Donkalnis ritual sites are embedded today. Accordingly, the multisensory experience of the place was interaction between us and the world as it is today (see Arlander 2012), and not with the Mesolithic–Neolithic landscape. Keeping in mind that we cannot escape our bodily experiences, we nonetheless captured and analysed our embodied experiences by using diverse media, some characterized by swiftness (digital methods) and others by a more gradual approach (analogue methods). Through these varied sensations, a combination of internal moods emerged, intertwined with the perceived place and landscape.

By focusing solely on the visual and spatial perception of the Spiginas and Donkalnis sites, we all concluded that the Spiginas site was a topographical anomaly that clearly stood out from the surrounding landscape. This was due to the impressive, steeply sloped hill located at the centre of the ancient island (Supplementary material 1 and 3). As this was also the location of the Mesolithic–Neolithic burials (Figure 4), the Spiginas site could easily be interpreted as a focal place marked with ritual deposits. Indeed, topographically, the Spiginas site was reminiscent of rock art sites, and although we could not explore whether the site had a clear echo during the Stone Age, the modern vegetation did create an echo effect. Consequently, the attributes of the Spiginas site seem to favour the agency of a place interpretation over the deathscape interpretation. This explanation is further supported by the scarcity of human burials over the course of several millennia (Table 1). In other words, the Spiginas site seems to be a topographically anomalous site that was set apart and marked with ritual deposits that took the form of human burials.

Compared to Spiginas, the Donkalnis site was visually less impressive: the slopes of the hill were not as steep as those of Spiginas and, overall, the former island was much smaller (Figure 3a; Supplementary material 1–2). However, as the ancient landscape was very clearly present at the site, it was evident that the Donkalnis site would have been visible from the nearby settlement site. Moreover, the former island was located very close to the shoreline in a shallow bay. Accordingly, the location would have been reachable not only by boat but also by swimming. According to Professor Butrimas, the seasonal changes in the water level might also have made the island a peninsula during times when the water levels were low (Butrimas, pers. comm.). Indeed, the long, narrow point of the former island extended very close to the shore and, on such occasions, could have acted as a kind of ‘avenue’ to the ritual site. Although no data are available for the seasonal water levels of Lake Biržulis, it is known that at least during the earlier part of the twentieth century the water level in the lake decreased, resulting in the discovery of Mesolithic artefacts and finds (Butrimas 2019, 193). If such seasonal changes were present also during the course of the Stone Age, such a phenomenon could have made the location anomalous or special in the eyes of the Mesolithic–Neolithic peoples. Indeed, events in which features become islands or are united with the mainland because of seasonal lake fluctuations would have affected the way people perceive and remember these places (Haskel and Stawski 2017). As Haskel and Stawski (2017) point out, such events would have been anticipated and resulted, for example, in the renegotiations of access to aquatic resources.

Here, it is important to note that a dynamic and changing coastline might also have had strong cosmological implications (Herva and Ylimaunu 2014). For example, in south-east Norway and Finland, processes of glacial retreat and sea-level changes have led to continuous land upheaval, that is, the emergence of new land from the sea. These changes pushed shore-bound Mesolithic and Neolithic settlements to the hinterland, while new settlements were established near the coast (e.g. Herva and Ylimaunu 2014; Nyland 2020a; Schülke 2020; Skantsi et al. 2023). Although the phenomenon has often been explained in terms of subsistence patterns and movement, it probably also impacted conceptions of the cosmos. As Herva and Ylimaunu (2014, 198) explain: ‘The dynamic nature of coastal landscapes – including, in particular, the emergence of new land from the sea – afforded symbolic and metaphorical links between landscapes and human life cycles. The perceived generation of new land from the sea meant that people could, and at least sometimes did, recognize that landscapes off the coast represented ancient coastal landscapes’. In this sense, it is interesting that the fourth millennium phase of use at Donkalnis coincides with a transgression of water levels – the exact opposite of emerging new land. Perhaps the disappearing coastlines also held similar symbolic importance as the emergence of a new land.

Intriguingly, the relationship between a dynamic coastal landscape and death is also evident with regards to the Late Mesolithic (*c.*6000–4000 Cal BCE) burial site of Skateholm II in southern Sweden (Figure 2), which was submerged due to marine transgression (Fahlander 2010). However, the burial activity at the site did not cease after this site was covered by water; rather, new burials were made near the submerged burial island (Larsson 1989). This suggests that the place was considered special and that people wanted to continue burying their dead at this location. Perhaps the changes in the coastline impacted these actions. Interestingly, something similar seemed to be going on also at the site of Jönsas (Figure 2) where water-polished cobbles, likely collected from the emerged ancient sea bottom, were commonly placed with the hunter-gatherer inhumations of the site, and, accordingly, might have served as mnemonic references to the ancient coastline (Ahola 2017, 107). In this light it seems reasonable to suggest that the dynamic coastline might have made the hunter-gatherer peoples dwelling in the ancient Lake Biržulis region mark the Donkalnis site with ritual deposits. In other words, the appearing and disappearing land might have given the Skateholm, Jönsas and Donkalnis sites an agency of their own.

Methodologically, our art/archaeology-influenced landscape analysis seemed to work well. We collected our perceptions individually and reflected upon the collected materials by discussing and comparing both visual and written notes. Regarding the artistic approach, the analysis of the visual photography material encompassed a comparison between moving digital images and still images and the analogue photographs in their various formats. Among these, the wide-angle digital panoramic photograph assumed the role of the most objectifying mode of representation, offering the most comprehensive view of the subjects (Supplementary material 1). Conversely, the square-format analogue photograph provided insights into the distinctive details of the places and generated partial compositions within the broader landscape. In black-and-white photography, both panoramic and square format, the apochronic (outsideness-of-time; Lassila 2023) aspect of the landscape was discernible, partly due to the monochromatic aesthetics and the timeless quality of film images (Supplementary material 1). The digital still image appeared the most documentary and perhaps the most commonplace of the explored alternatives. The advantage of moving images was the ability to approach the ground level and come closer to sensory experience and also to document the soundscapes of the sites (Supplementary material 2–3). The videographs also proved to be the best way to document the topography of the sites. This was especially true for the Spiginas site (Supplementary material 3), which was difficult to document with still images or drawings due to the dense forest.

As expected, the drawings made on the sites (Figure 5) acted as visual notes of our perceptions. However, combined with the traditional written field notes, the kinetic experience of moving around the site and copying the bones of the landscape onto paper also enforced the visual and spatial perception of the landscape and, accordingly, allowed us to pay attention to all visible details. In other words, drawing at the sites clearly forced us to slow down and concentrate on the perception of the landscape. When using analogue photography techniques, similar slowing down and concentrating on the subject in front of us was necessary. The limited amount of film negatives (12 negatives on medium-format film used by the Rolleiflex) forces the photographer to stop, perceive and compose more carefully than with a digital camera capable of capturing several images per second and storing up to 37,600 images on a single memory card. Considering that we were not able to return to the site repeatedly, we all agreed that this ‘doing landscape’ – whether through camera lens or drawing – was beneficial to our analysis. Indeed, we argue that this practice created what Arlander (2012) considers ‘mementos of moments’ that we could reflect upon after the fieldwork. Simultaneously, the photographs and drawings can be understood as the material remains of our ‘sensuous scholarship’ (Skeates and Day 2020).

Remarkably, the artist of the team paid attention to the same landscape features as the archaeologists, suggesting that these attributes were not entirely influenced by archaeological knowledge. In fact, according to the tacit knowledge collected during the fieldwork, local archaeologists and laypeople living in the region noted the same topographical anomalies as we did. Accordingly, even though we did not visit sites with different age groups, sexes and genders (see Brück 2005), the people and bodies reached within this research suggest that the perception of a topographic anomaly is shared among different people. However, in the future, it might be beneficial to bring a larger, heterogeneous group of people to the sites (e.g. Hamilton et al. 2006). Then, we could also further test the ‘doing landscape’ approach by giving some people the opportunity to draw or take analogue photographs while others would document their embodied experiences solely with written notes.

## 5. Conclusions

During the Mesolithic and Neolithic, the foragers dwelling in the Eastern Baltic, Scandinavia and Fennoscandia regions deposited some of their dead underground (e.g. Ahola 2019; Butrimas 2012, 2019; Larsson 1989; Tõrv 2016; Zagorskis 2004). As many of these burial sites are located on lake islands or other coastal sites, a strong connection between death and water has been suggested (Butrimas 2012; Zvelebil 2003). However, when explored from the perspective of relational archaeology, these sites could be more than ‘deathscapes’, that is, places where landscape features are solely associated with death. Indeed, when rejecting classic ‘humanistic’ divides such as nature–culture, human–animal and animate–inanimate, and accordingly living–dead, and instead accepting the agency of both humans and other-than-human agents (e.g. Harrison-Buck and Hendon 2018; Herva and Lahelma 2020), these locations could also be understood as social agents and the human burials as means of communication with these agents.

Our novel analysis of the Spiginas and Donkalnis Mesolithic–Neolithic ritual sites in western Lithuania brought to light attributes that might have given these sites social agency of their own and, accordingly, contributed to the ways in which ancient foragers visited, revisited and marked these locations with depositional acts that took the form of human burials. Indeed, as our sensory and artistic approach to landscape showed, the former island of Spiginas was an easily recognizable topographical anomaly that likely demanded attention during the Mesolithic and Neolithic Stone Age. However, as burials were made there only rarely, it is likely that the site was not considered a place of burial; instead, the burials were made to be part of the place. In this sense, the location should be understood not solely through ideas relating to death and afterlife, but instead in the way hunter-gatherer peoples dwelling in the region communicated with the surrounding world.

In contrast to the Spiginas site, the former island of Donkalnis was less visible with more human burials. As on Spiginas, however, these depositional acts were performed only rarely and with long hiatuses between deposits. According to our analysis, the site was nonetheless a peculiarly shaped location that easily caught the eye: it was visible from several directions and marked the access to the shoreline. Indeed, located in a shallow bay, near a settlement site with a Stone Age date, the island was a place that one would naturally pass. It was also easily accessible from the shore, and due to seasonal differences in the water levels of the ancient Lake Biržulis, the island might occasionally have been a peninsula to which people could have walked. Although no exact data exist of the seasonal changes, lake transgressions and regressions were typical for the ancient Lake Biržulis during the fourth millennium BCE (Butrimas 2019, 195). Such dynamic change in the landscape and the place itself might have given Donkalnis its agency. In other words, the depositional acts could have been conducted at the site due to the changing nature of the place.

In conclusion, when the perspective changes from ‘deathscapes’ to a more relational view of landscape as a social agent, new insights into Mesolithic–Neolithic considerations of the cosmos can be gained. Indeed, when attention is paid to the sensory attributes and changes in the landscape of the burial sites and human burials are viewed as depositional acts instead of ritual responses to death, a more nuanced view of ancient depositional practices emerges. In light of our research, it seems reasonable to assume that the hunter-gatherer peoples of the ancient Lake Biržulis region buried a small proportion of their dead at focal points in the landscape, perhaps to mark the location, to be part of the place itself or to communicate with the surrounding world, while most of the community were subject to completely different mortuary practices. In other words, inhumation might not have been considered a normative way to dispose of a dead body, as it is today. Instead, the dressed and decorated dead human body, an anomaly itself, might have been considered a powerful tool to negotiate with the non-human agents dwelling in the world.

## Supplementary Material

Supplemental Material

Supplemental Material

Supplemental Material

## Note

1. Supplementary material can be accessed here: https://doi.org/10.5281/zenodo.8409340
