# Supplementary material for: Doing landscape: sensorial and artistic approaches to Donkalnis and Spiginas Mesolithic–Neolithic ritual sites in western Lithuania
Source: Time Mind. 2024 Apr 24;17(1-2):9–33. doi: 10.1080/1751696X.2024.2338055 (PMC11404861; doi:10.1080/1751696X.2024.2338055)
Supplement: Supplemental Material [file RTAM_A_2338055_SM4729.pdf]

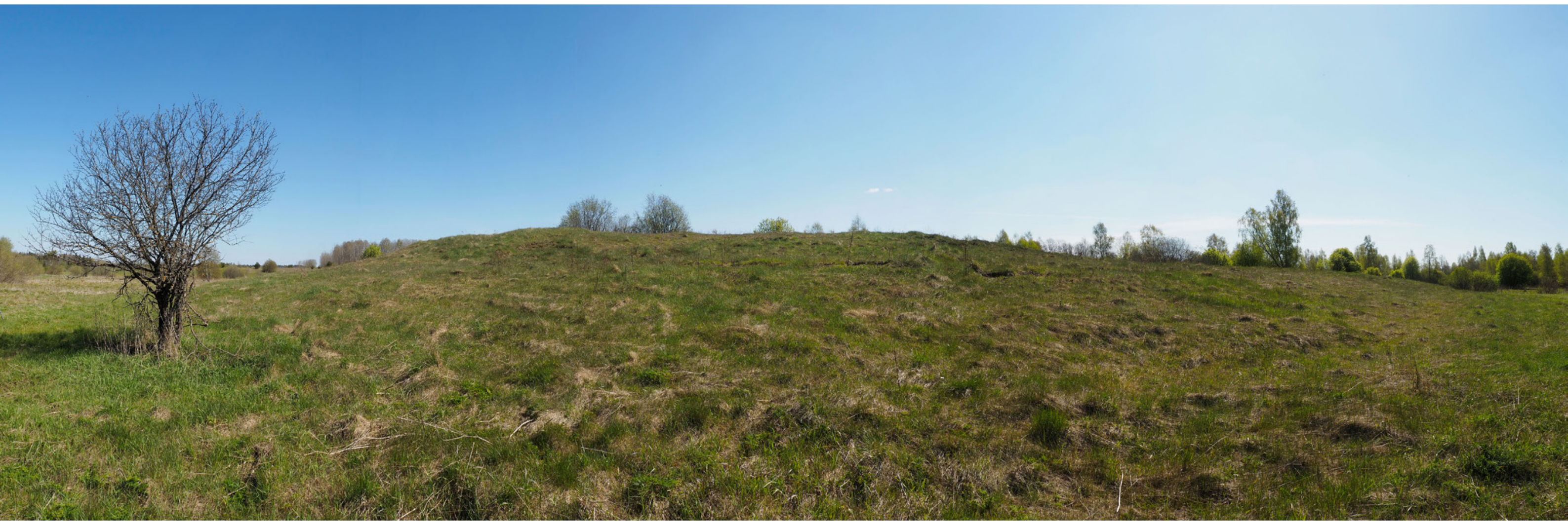
1.

Views Over Time

2023

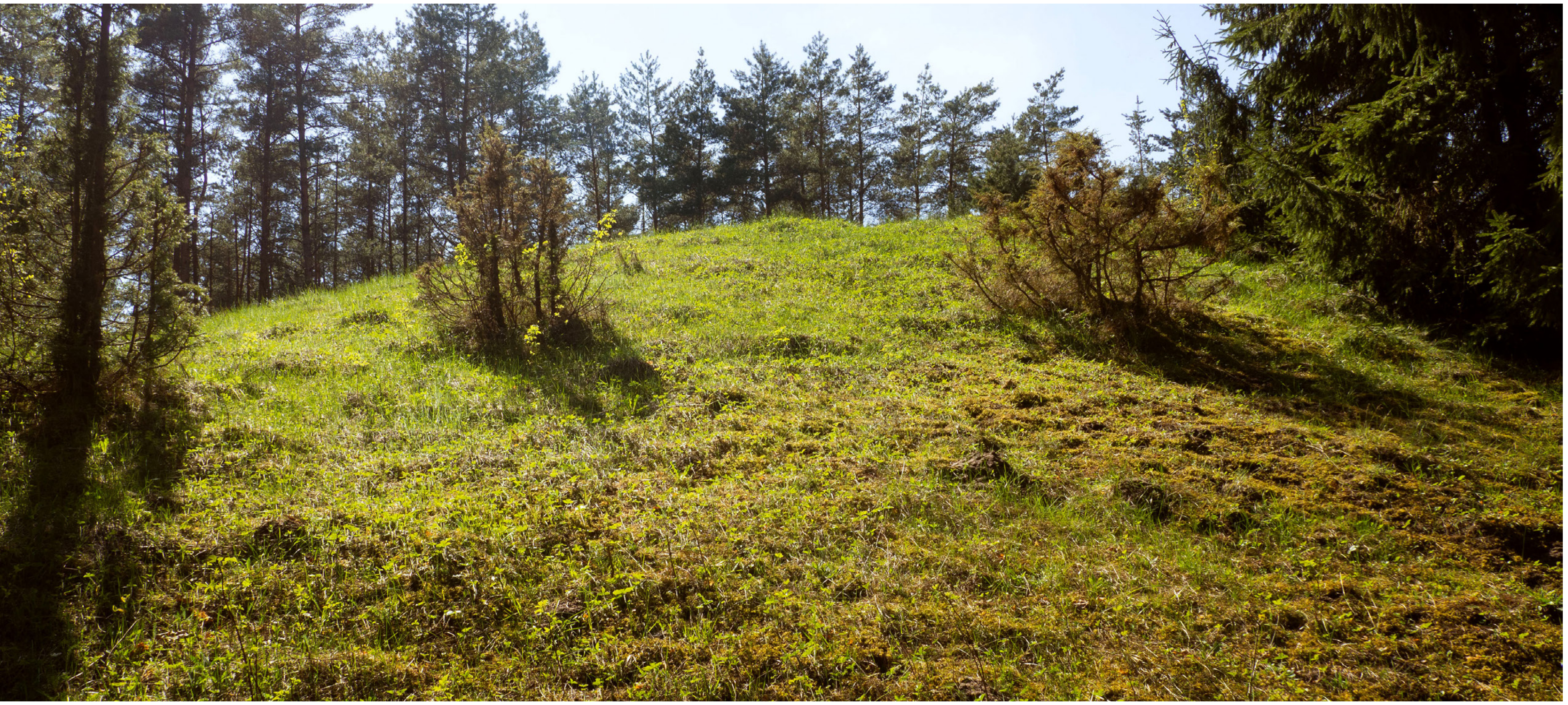
2.

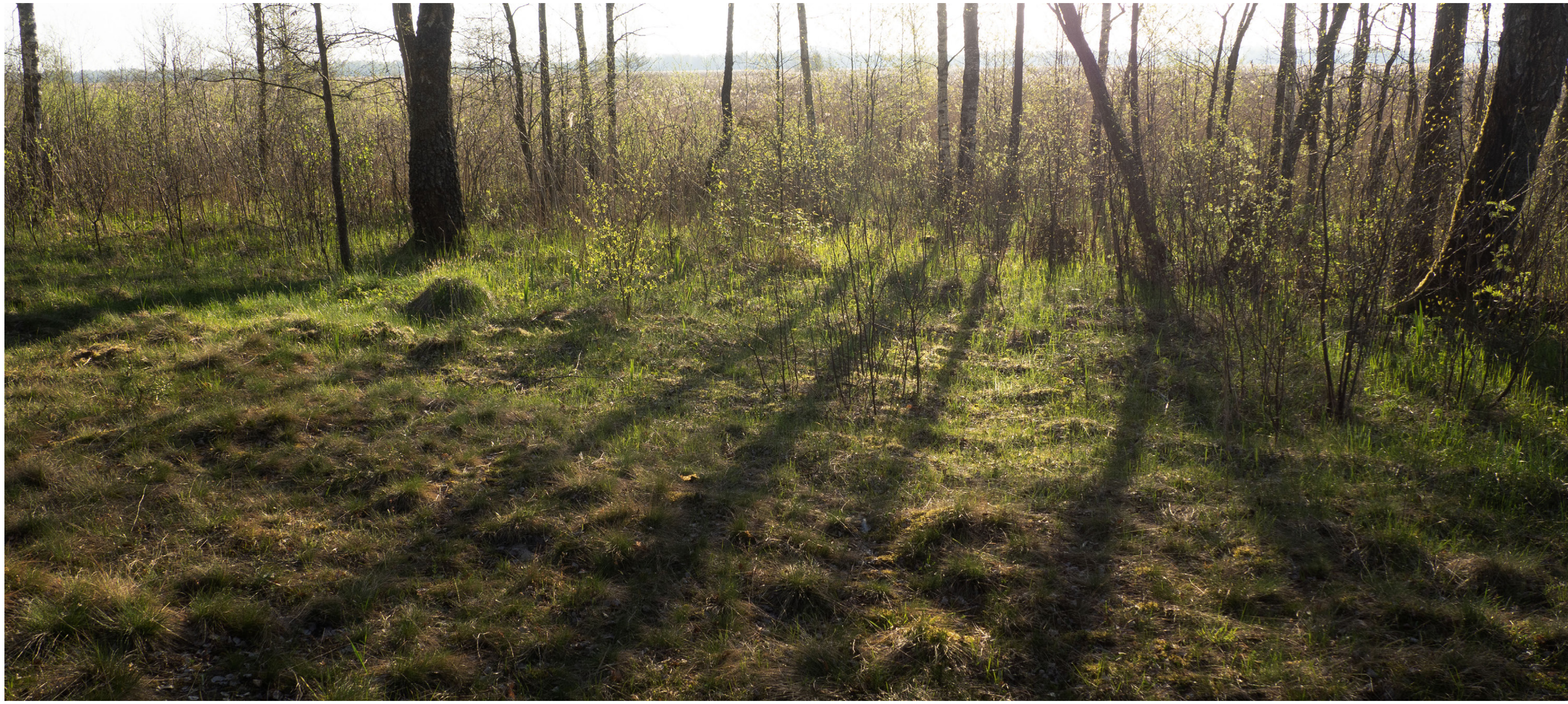
3.

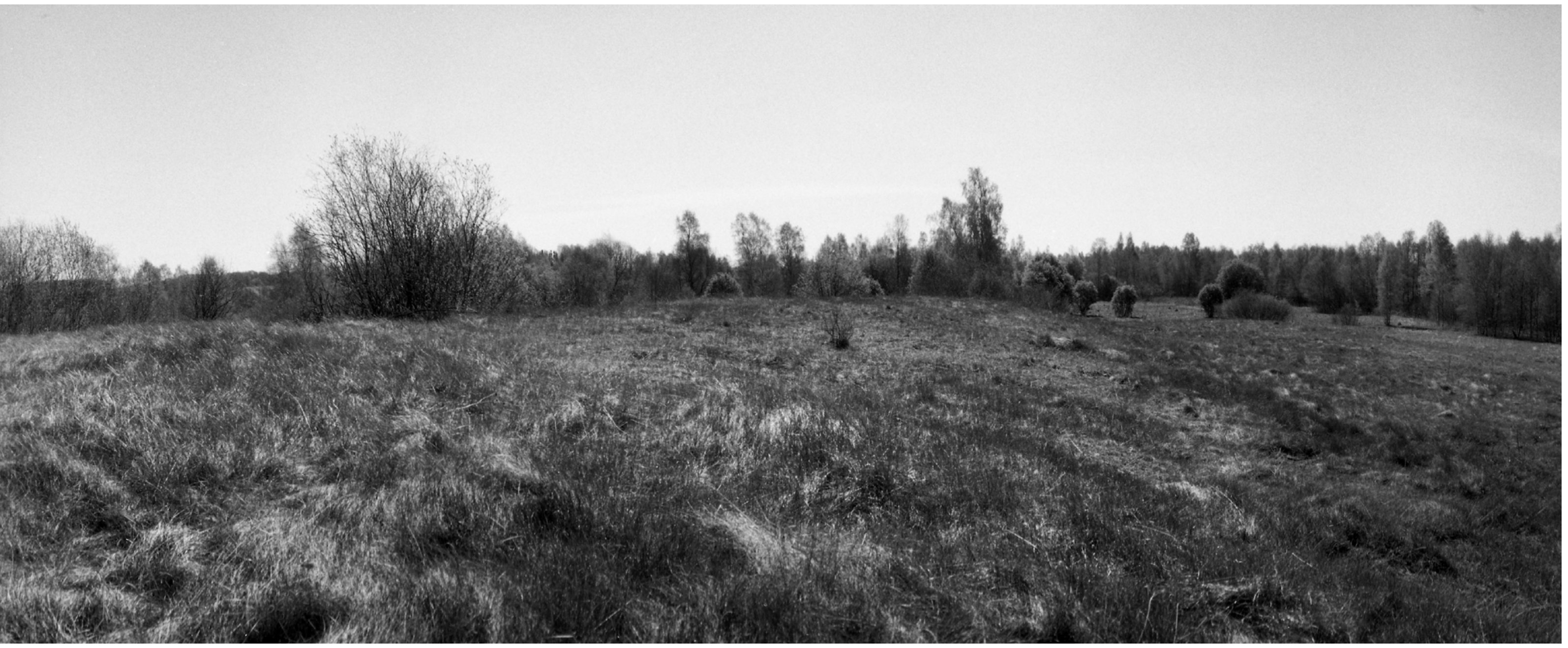

4.

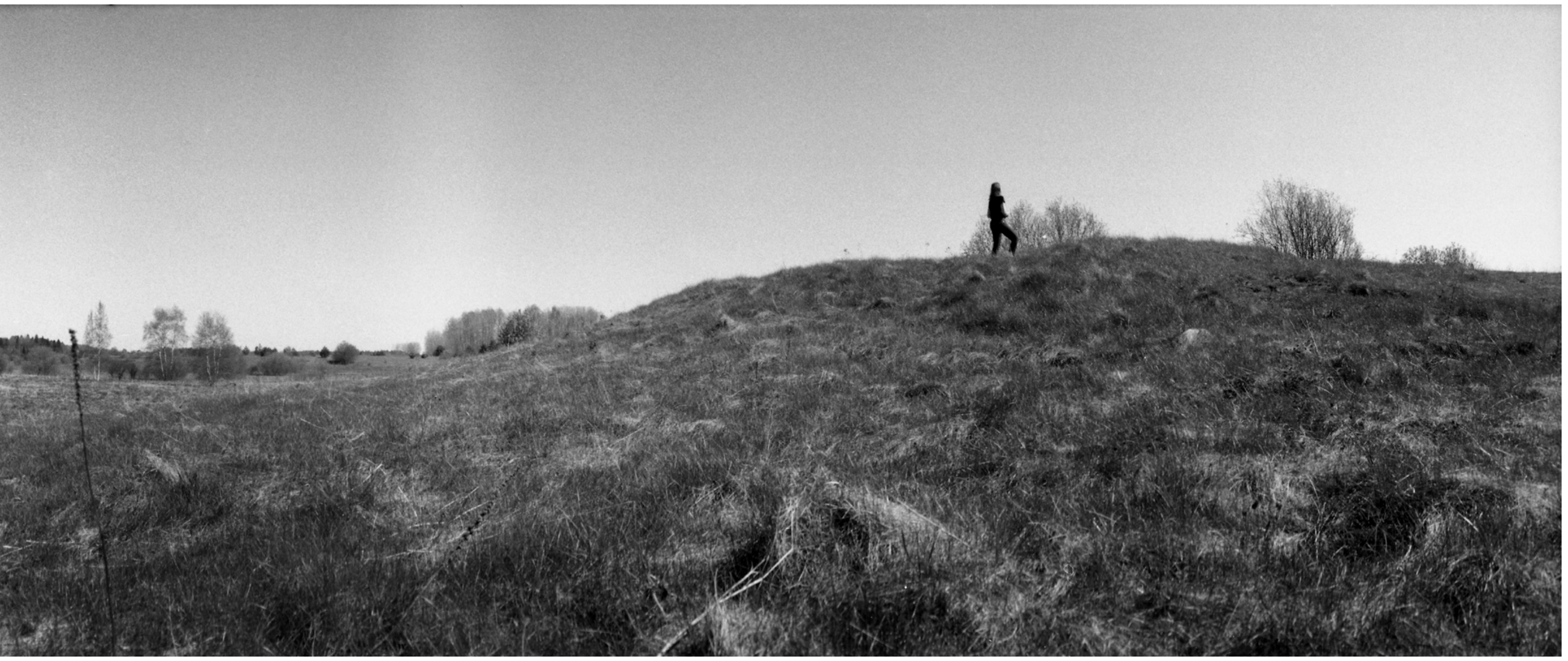

5.

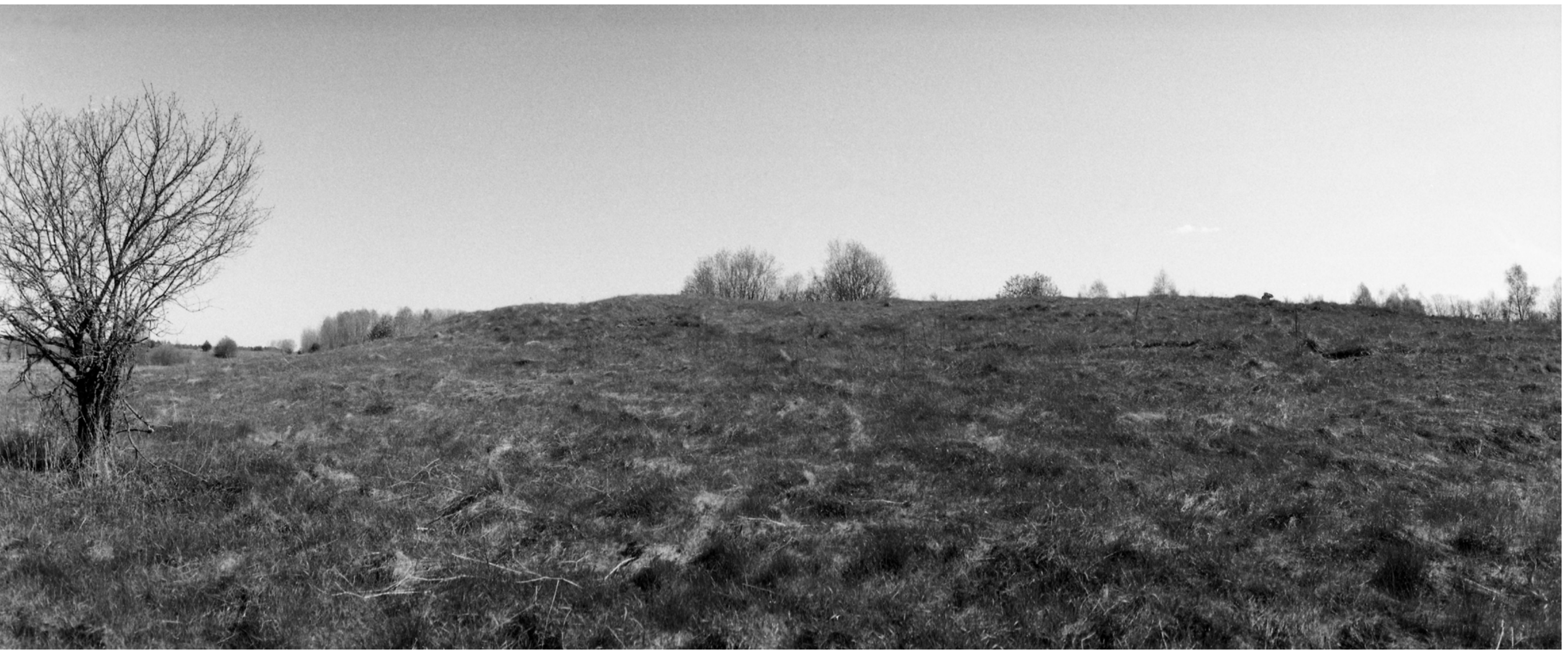

6.

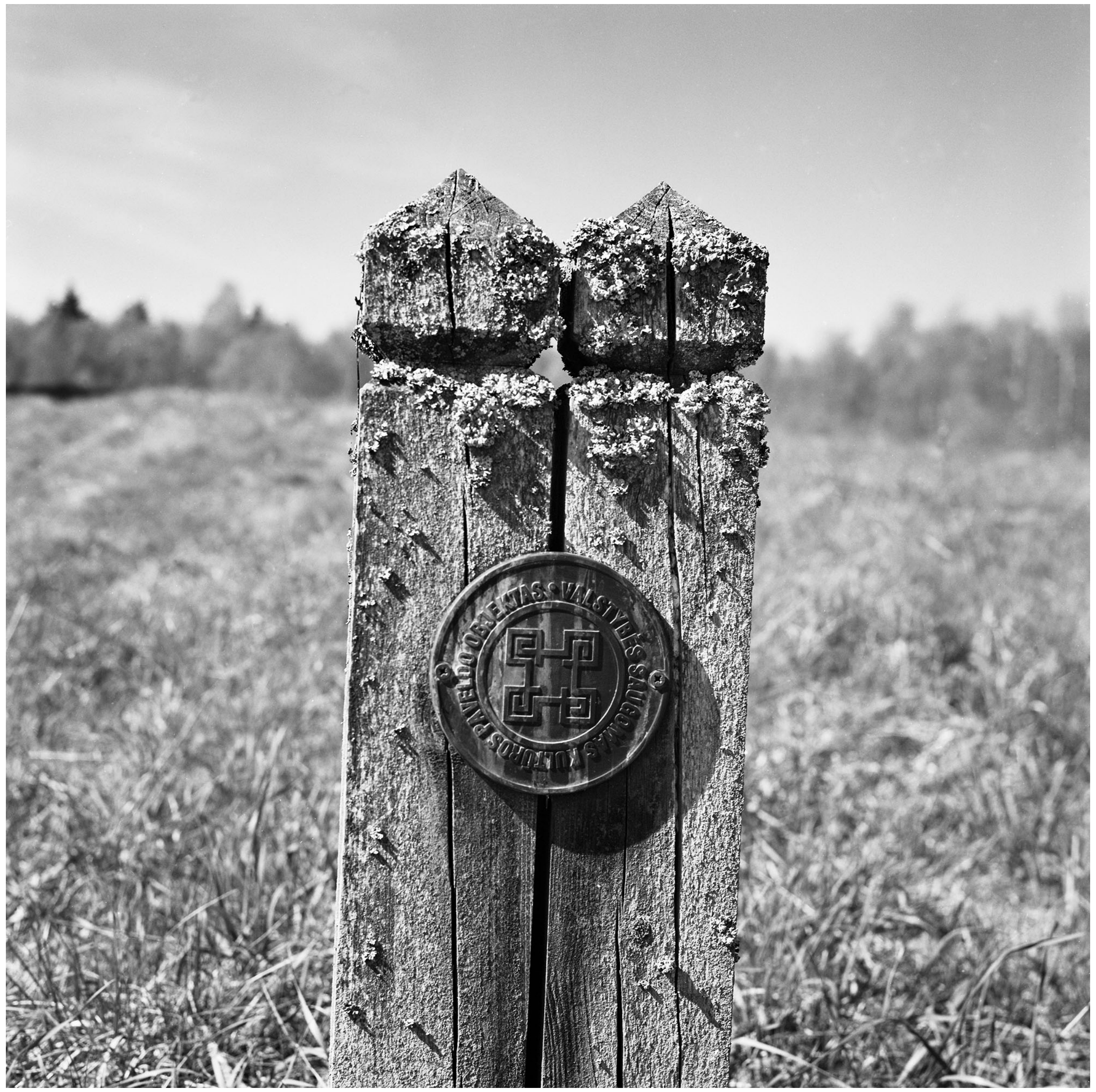

7.

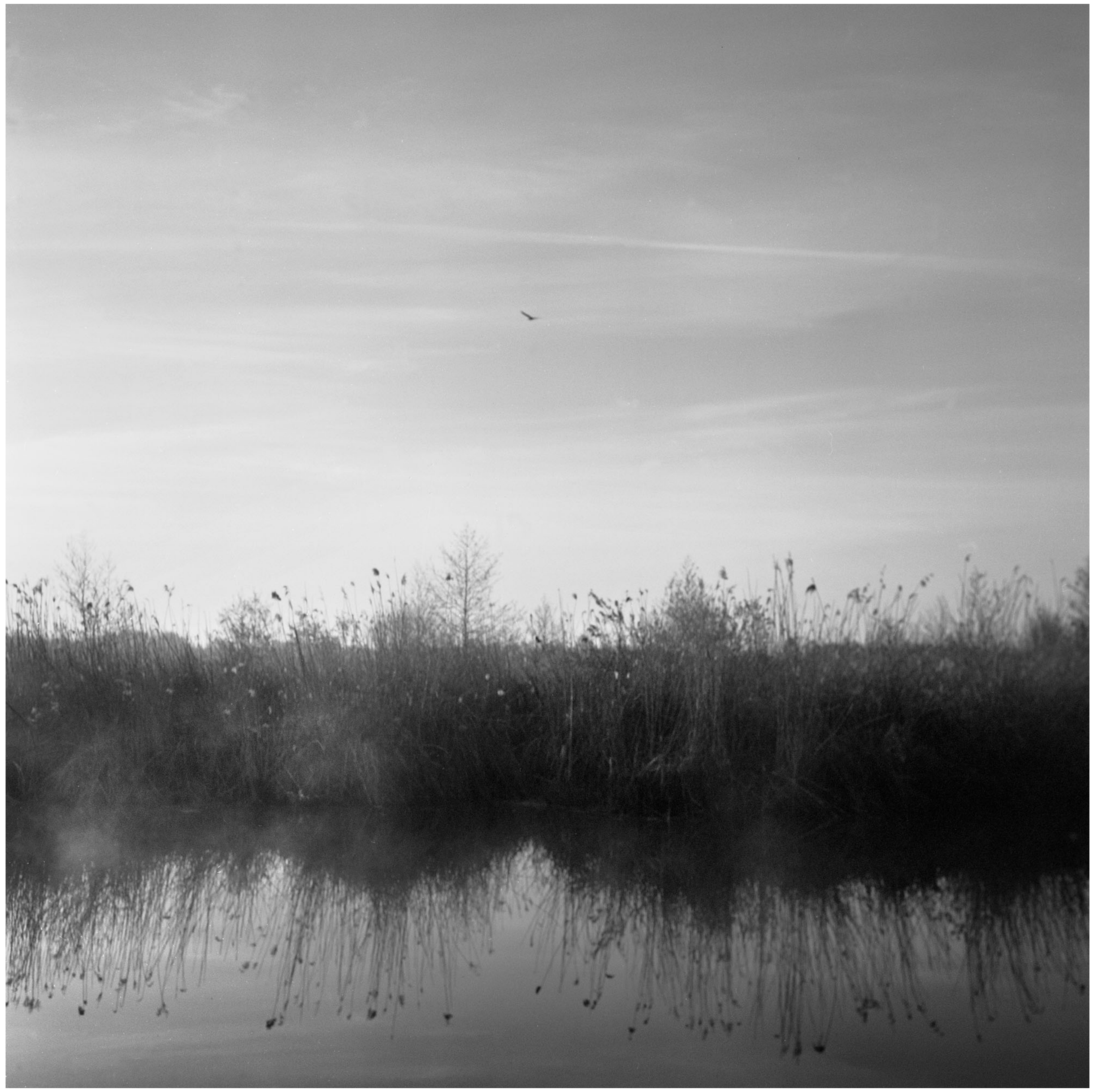

8.

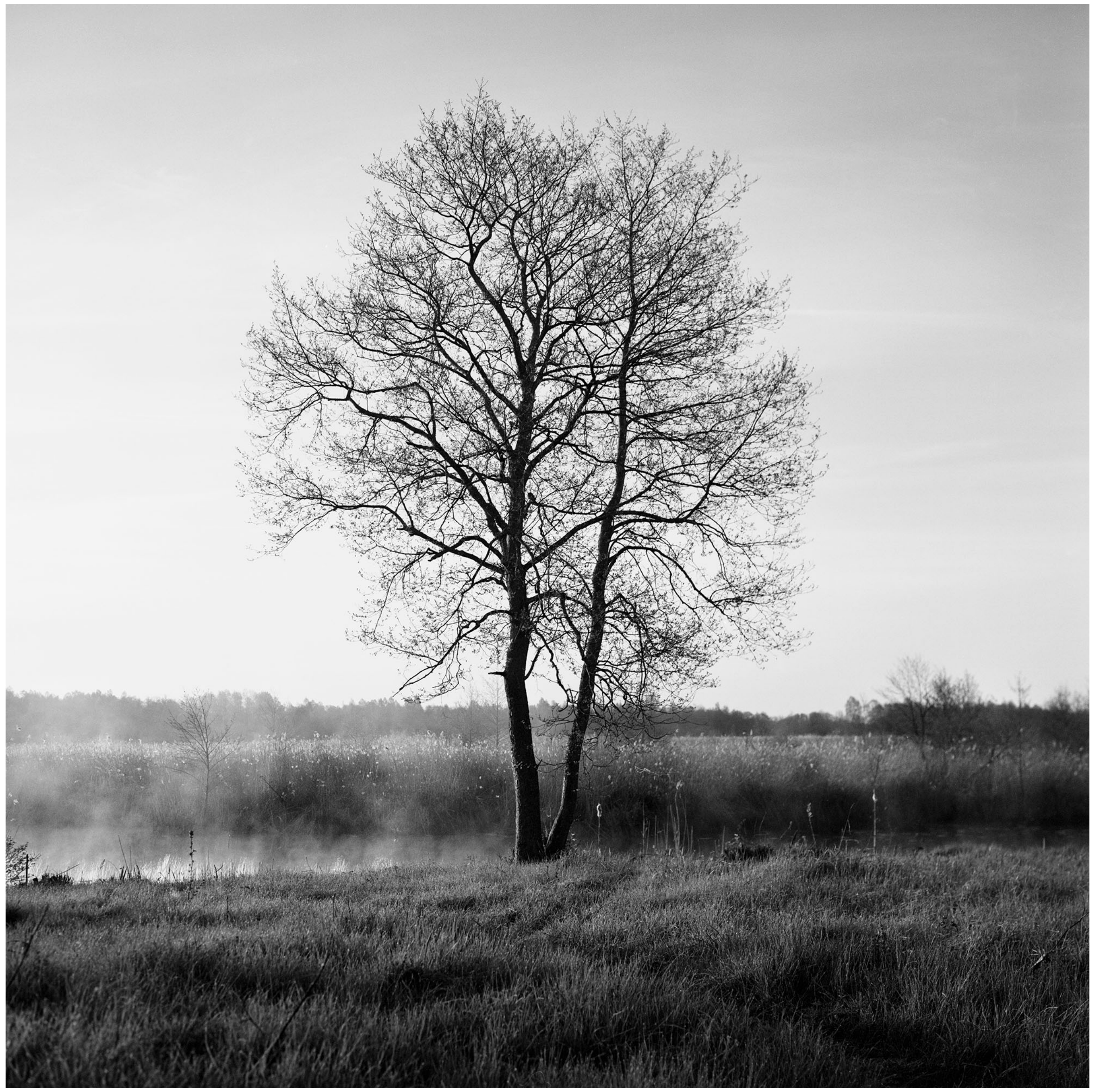

9.

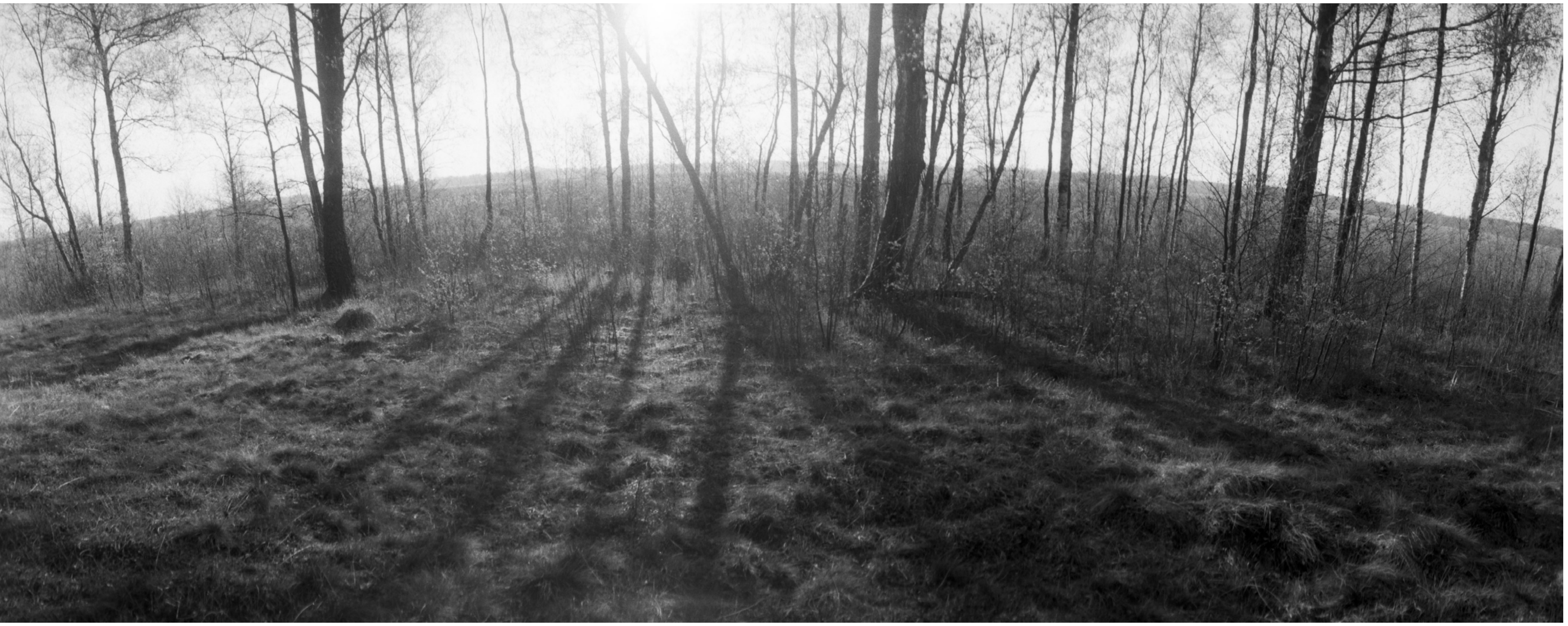

10.

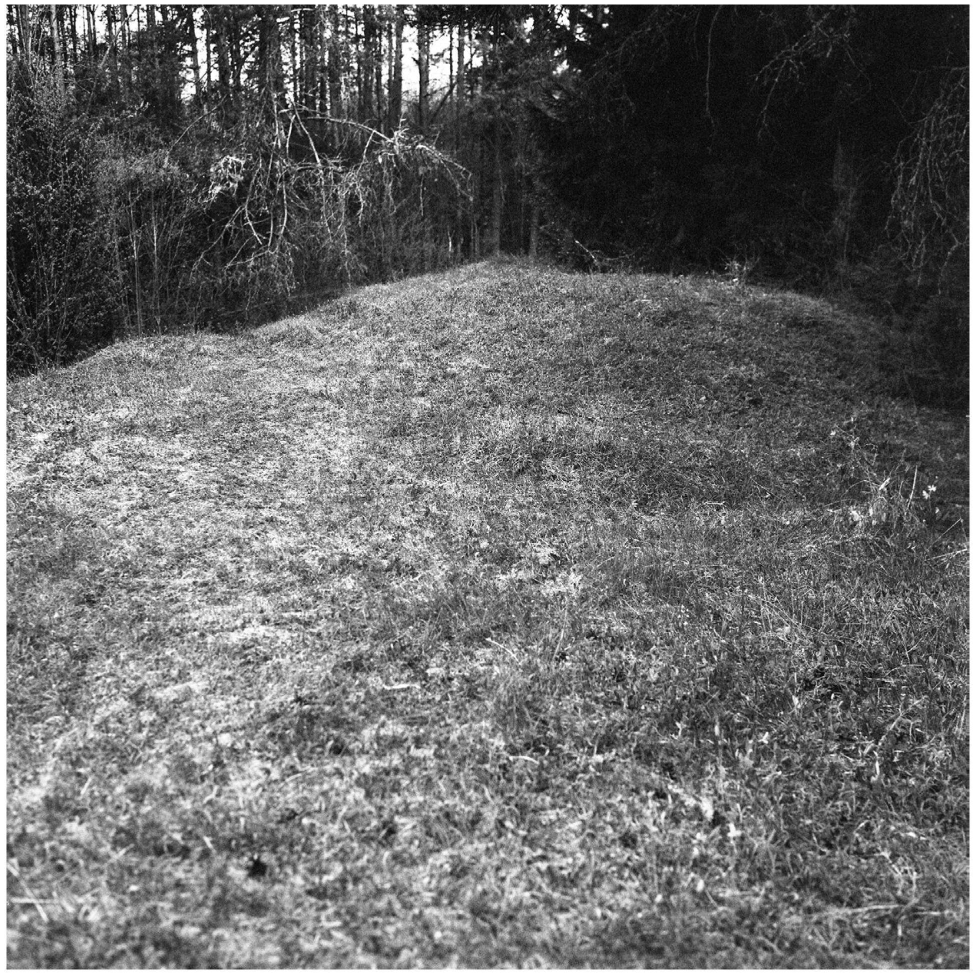

11.

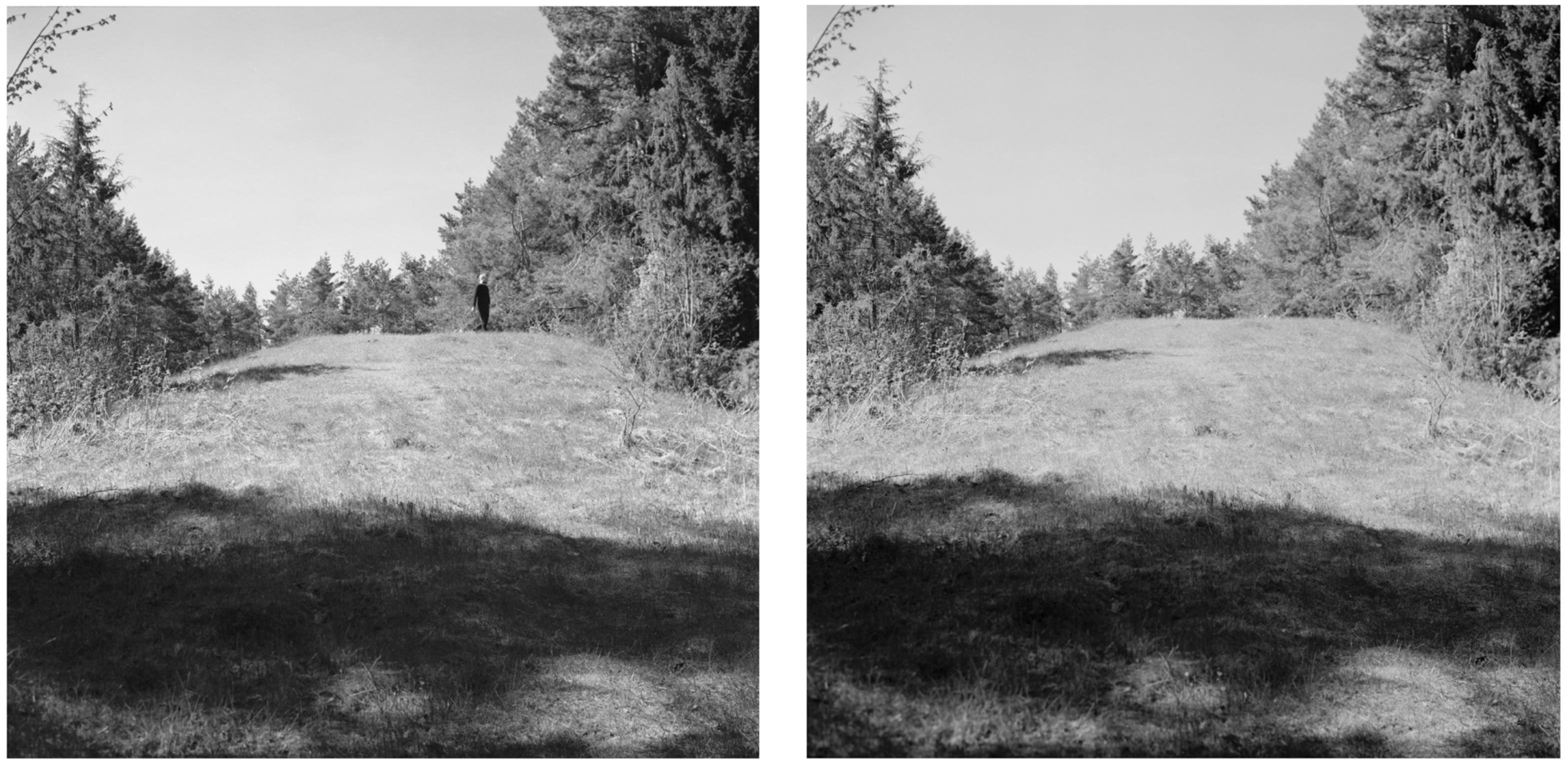

12.

List of Photographs

Photograph 1. Digital photograph of Donkalnis site. Image composite of 5 different pictures.

Photograph 2. Digital photograph of Spiginas site.

Photograph 3. Digital photograph of the ancient shoreline, near Spiginas site.

Photograph 4. Donkalnis panorama from the top of the site. Film photograph taken with panoramic Horizon camera.

Photograph 5. Donkalnis panorama to the site with a human being. Film photograph taken with panoramic Horizon camera.

Photograph 6. Donkalnis panorama to the site. Film photograph taken with panoramic Horizon camera.

Photograph 7. Donkalnis site archaeological site sign. Film photograph taken with medium format Rolleiflex camera.

Photograph 8. Lake Biržulis reflections. Film photograph taken with medium format Rolleiflex camera.

Photograph 9. A Tree at the Lake Biržulis. Film photograph taken with medium format Rolleiflex camera.

Photograph 10. Trees at the ancient shore line near Spiginas site. Film photograph taken with panoramic Horizon camera.

Photograph 11. View from the top of the Spiginas site. Film photograph taken with medium format Rolleiflex camera.

Photograph 12. A Diptych of the Spiginas, with and without a human being. Two film photographs taken with medium format Rolleiflex camera.
